# Maternal Nutrition and Gestational Weight Gain Among Saudi Women: Riyadh Mother and Baby Follow Up Study (RAHMA Explore)

**DOI:** 10.3390/healthcare13121446

**Published:** 2025-06-16

**Authors:** Hayfaa Wahabi, Amel Fayed, Samia Esmaeil, Ansam Ayman Almadhun

**Affiliations:** 1 Research Chair for Evidence-Based Health Care and Knowledge Translation, King Saud University, Riyadh 11451, Saudi Arabia; hwahabi@ksu.edu.sa (H.W.); sesmaeil@ksu.edu.sa (S.E.); 2 Department of Family and Community Medicine, College of Medicine, King Saud University, Riyadh 11451, Saudi Arabia; 3 Department of Family and Community Medicine, College of Medicine, Princess Nourah Bint Abdulrahman University, P.O. Box 84428, Riyadh 11671, Saudi Arabia; 4 Department of Nursing Services, King Khalid University Hospital, Riyadh 12372, Saudi Arabia; aalmadhun@ksu.edu.sa

**Keywords:** FIGO nutrition checklist, maternal nutrition, gestational weight gain

## Abstract

**Background:** Maternal nutrition is one of the main determinants of healthy pregnancy outcomes. The aim of this study is to investigate maternal nutritional risks and their relationship with gestational weight gain (GWG) among Saudi women. **Methods:** This is a cross-sectional study conducted in the antenatal clinics of a university hospital. The FIGO Nutrition Checklist was used to investigate the nutritional habits of pregnant women attending their regular antenatal visits. The FIGO tool includes a brief food frequency questionnaire (FFQ) and total nutritional risk score (NRS). Data on other variables were collected, including participant demographics and obstetric history. Current weight and height were extracted from nursing notes of the current visit, and the pre-pregnancy weight was self-reported by participants. GWG was reported and participants were classified according to IOM Guidelines. All data were analyzed using SPSS (Version 30, release September 2024) and *p* < 0.05 was defined as statistically significant. **Results:** A total of 570 pregnant women participated in the study, of whom 96% had at least one nutritional risk. More than 90% of participants reported sufficient folic acid intake, normal hemoglobin level and adequate meat and poultry intake. Only 23.9% of participants had sufficient fish intake and 24.6% reported proper sun exposure. Additionally, 10% of participants scored poorly on the FFQ, while 30% were classified as high-risk based on NRS scale. Poor nutritional scores were not associated with any clinical or socioeconomical variables. According to IOM guidelines, 26.3% of the participants achieved adequate GWG, while 49.5% had inadequate GWG, and 24.2% exceeded recommended GWG. Inadequate GWG was most common among those with low pre-pregnancy BMI (60%), followed by overweight (43.2%) and obese (37%) women. Neither parity nor nutritional scores significantly influenced GWG. **Conclusions:** Although poor nutritional quality and high nutritional risk are relatively uncommon among Saudi women, the prevalence rates remain consistent across all sociodemographic groups. This suggests widespread, uniform patterns of suboptimal dietary habits within the community. While GWG was not affected by nutritional status or parity of the participants, nearly half of participants had inadequate GWG, particularly those with a low pre-pregnancy BMI.

## 1. Introduction

Maternal nutrition is a key determinant of healthy pregnancy outcomes [1] and long-term child and adult health [2,3]. Both undernutrition and obesity during pregnancy can lead to adverse outcomes, with lasting effects on offspring. Maternal obesity and high-calorie diet increase the risk of childhood and adult obesity [4], as well as stroke, coronary heart disease and type 2 diabetes [5]. On the other hand, maternal suboptimal nutrition is associated with infants that are small for their gestational age, low birth weight and with microcephaly [6]. Additionally, poor maternal nutrition may predispose offspring to metabolic disorders later in life, including hypertension, dyslipidemia, impaired glucose tolerance, and overweight/obesity, hence, increasing the risk of non-communicable disease [7,8].

The rising prevalence of obesity among women of reproductive age, coupled with maternal malnutrition—particularly iron deficiency anemia [9,10,11]—has made maternal nutrition a priority for scientific societies. This has led to the development of nutritional assessment tools and guidelines for appropriate Gestational Weight Gain (GWG). The International Federation of Gynecology and Obstetrics (FIGO) Nutrition Checklist is one such tool, designed to detect dietary imbalances during preconception, pregnancy, and postpartum periods [12]. Moreover, it offers valuable feedback and counseling to women regarding their dietary habits and nutritional gaps [12].

Current research clearly indicates that maternal obesity is the primary contributor to adverse pregnancy outcomes among Saudi women [13,14,15]. Most of the studies examining maternal weight and its effects on pregnancy outcomes were published from the Riyadh Mother and Baby multicenter study (RAHMA) database. RAHMA is the largest longitudinal cohort study conducted in Saudi Arabia [16]. The database of the study includes antenatal, perinatal, and postnatal data on 14,568 women and their offspring [16].

RAHMA study revealed that over 68% of pregnant women in Saudi Arabia were obese or overweight before pregnancy [13], which significantly increased their risk of developing gestational diabetes (GDM), high blood pressure during pregnancy, cesarean section delivery, and delivering macrosomic infants [13,17]. Longitudinal follow-up of this cohort demonstrated that 35% of participants retained more than 7 kg of GWG one year postpartum [15]. Notably, this excessive postpartum weight retention (PPWR) was associated with adverse cardiometabolic outcomes, including hyperglycemia and metabolic syndrome [15]. To better understand the determinants of maternal obesity among Saudi mothers, a series of follow-up studies, under the name of RAHMA (explore), were conducted, which includes the current study.

The objectives of this study are:

1. To investigate the nutritional status and the nutritional risks of Saudi pregnant women during the three trimesters of pregnancy using the FIGO nutritional checklist.
2. To investigate the effect of the nutritional risks on GWG.

## 2. Methods

### 2.1. Study Design and Setting

This is a cross-sectional study conducted in the antenatal clinics of a University Hospital in Riyadh, Saudi Arabia. The study was designed to investigate the influence of women’s nutritional habits on GWG.

### 2.2. Study Participants

Women attending antenatal clinics for their regular visits were invited to participate in the study after clarifying the objectives of the study. All women were eligible to participate in the study if they were able to sign the consent for participation. There was no restriction to a certain stage of pregnancy or any comorbidity except for women with bariatric surgery or illnesses which restrict food intake or who suffer from chronic illnesses such as sickle cell anemia or autoimmune diseases.

### 2.3. Sampling Technique and Sample Size

A purposive non-probability sampling technique was adopted in this study. As we are planning to investigate the nutritional status of pregnant women, we based our priori sample size calculation on estimation of prevalence (effect size) of good nutritional habits among pregnant women. We were expecting that the good nutritional habit prevalence would be around 50% (effect size/margin of error = 10%) with a level of confidence of 95% (alpha 5%) and power of 80% (Beta 20%); the minimal sample size was 504, as calculated by G-Power software, version 3.1.

### 2.4. Study Tool

To investigate the nutritional habits of participants, we used the FIGO Nutrition Checklist, which is a validated tool designed to identify imbalanced diets during preconception, pregnancy, and postpartum periods. The FIGO Nutrition Checklist gathers information on dietary practices, including special diets, body mass index, diet quality (measured by the frequency of consuming specific foods), and micronutrient intake such as folic acid, vitamin D, and iron.

Questions on the FIGO nutrition checklist were divided into two sections. The first section included six dietary intake questions designed as a brief food frequency questionnaire (FFQ). These questions were utilized to compute the FIGO-diet quality score; each affirmative response (Yes) to the six FFQ questions was assigned one point, while negative responses received zero points. The total diet quality score was derived by summing these points, with the score (a continuous variable) ranging from 0 to a maximum of six, where higher scores indicated better diet quality. For this study a score ≤3 is considered a poor FFQ score. Concurrently, the same six dietary intake questions and three questions on oral nutrient supplementation of the checklist were used to calculate the total nutritional risk score (NRS) based on the FIGO nutrition checklist. The FIGO-NRS ranges from 0 to 9, with lower scores indicating a higher number of nutritional risk factors. For this study a score of ≥5 is considered a low nutritional risk score (good nutritional status).

The original version was in English; the Arabic version is available from the official FIGO website for download and use [18]. The Arabic version was revised by a group of Saudi women to confirm the clarity of all questions, and no further modifications were recommended. Piloting of the questionnaire was performed among 12 participants to confirm feasibility and clarity of all items.

Data on other variables were collected including participant demographics (age, marital status, educational attainment, and income) and obstetric history (parity, trimester of pregnancy, presence of hyperemesis gravidarum, GDM, and diagnoses of preeclampsia or hypertensive disorders of pregnancy). Current weight and height were extracted from nursing notes of the current visit, and the pre-pregnancy weight was reported by participants.

### 2.5. Definitions

**Pre-pregnancy BMI:** According to pre-pregnancy BMI, participants were categorized into 4 groups: underweight (BMI < 18.5 kg/m^2^), normal weight (BMI = 18.5–24.9 Kg/m^2^), overweight (BMI = 25–29.9 Kg/m^2^) and obese (BMI ≥ 30 kg/m^2^).

Pre-pregnancy weight was self-reported by participating women; self-reported pre-pregnancy weight remains the most common measurement in clinical practice. Importantly, research demonstrates that self-reported pre-pregnancy weight obtained during pregnancy shows good reliability and validity.

**Gestational weight gain** was calculated as the difference between the current weight and the pre-pregnancy weight.

Institute of Medicine guidelines (IOM) for proper GWG according [19] to pre-pregnancy BMI: This classification was applied only to women who were in the third trimester near their full term. According to the IOM guidelines, proper GWG for underweight women is in the range of 12.5–18 Kg, for normal weight 11.5–16 Kg, for overweight 7–11.5 Kg and for obese 5–9 Kg. Inadequate GWG was defined if women did not reach the proper GWG, while excessive GWG was considered if the participating women exceeded the proper range as defined by IOM.

**GDM** is diagnosed at any time in pregnancy according to World Health Organization guidelines if one or more of the following criteria is met [20]:

- Fasting plasma glucose 5.1–6.9 mmol/L (92–125 mg/dL);
- 1 h plasma glucose ≥10.0 mmol/L (180 mg/dL) following a 75 g oral glucose load;
- 2 h plasma glucose 8.5–11.0 mmol/L (153–199 mg/dL) following a 75 g oral glucose load.

**Pre-gestational Diabetes Mellitus (PGDM)** is a condition in which the mother has diabetes (most commonly type 1 or type 2 diabetes) before the onset of pregnancy

**Hypertensive events during pregnancy** according to the report on national high blood pressure [21]: Pre-eclampsia is defined as new onset of elevated blood pressure after 20 weeks of pregnancy in a previously normotensive woman (≥140 mm Hg systolic or ≥90 mm Hg diastolic in addition to proteinuria of at least 1+ on a urine dipstick or ≥300 mg in a 24 h urine collection). Gestational hypertension is defined as new onset of elevated blood pressure (≥140 mm Hg systolic or ≥90 mm Hg) after 20 weeks of gestation in a previously normotensive woman and superimposed pre-eclampsia as new onset of pre-eclampsia after 20 weeks of pregnancy.

### 2.6. Statistical Analysis

Data entry was performed using Research Electronic Data Capture (RED-Cap), which is an electronic data capture tool hosted at Princess Nourah bint Abdulrahman University (PNU). RED-Cap is a secure, web-based software platform designed to support data capture for research studies. SPSS (IBM Corp. Released 2016. IBM SPSS Statistics for Windows, Version 24.0. Armonk, NY, USA: IBM Corp.) was used for data analysis. Description of categorical variables was carried out in terms of frequency and percentage, while quantitative variables were expressed using median and interquartile range (IQR). The Chi-square test was used to test the association of categorical variables. Mann–Whitney U test and Kruskal–Wallis test were used to compare GWG among different groups. A generalized linear model was adopted for testing the relation between GWG (as the response variable) and NRS adjusted for trimester of pregnancy, parity, maternal age, GDM and hypertension during pregnancy. As GWG was not normally distributed, the generalized linear model was chosen using Gamma with log link type, the AIC and BIC were the least for the selected model and the goodness of fit test was statistically significant (likelihood ratio chi-square = 71.5, degree of freedom 7 and *p*-value < 0.001).

### 2.7. Ethical Consideration

The study followed the principals of the Helsinki declaration and was approved by Princess Nourah Bint Abdulrahman Institutional Review Board (IRB log number 23-0203, 12 April 2023). Informed consent was provided by all participants before participation in the study.

- Informed Consent Statement

After providing a clear explanation of the study’s objectives, all participants gave their consent. Participation in the study was completely voluntary, and participants were informed that they could withdraw at any time upon request.

## 3. Results

A total of 570 pregnant women, who were attending the clinics for their regular antenatal care, consented to participate in the study. Approximately two-thirds of the participants were between 20 and 35 years of age, with only five women below the age of 20. The educational background of the participants varied, with nearly 60% being university graduates. Around 65.8% were housewives, and more than 80% reported having sufficient family income. A small proportion of participants were in their first trimester (13.7%), while the majority were in their third trimester (53.0%). Only 38.4% of the women had a normal BMI. Hyperemesis gravidarum was reported in 1.2%, hypertension in 2.1%, diabetes in 3.9%, and 11.9% suffered from GDM (Table 1).

**Figure 1:**
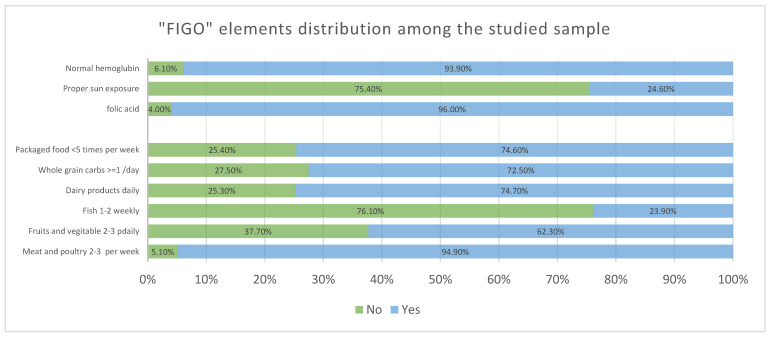
Distribution of FIGO elements among the participants in the study (*n* = 570).

The distribution of all FIGO checklist elements is shown in Table 2 and Figure 1. Sufficient folic acid intake, normal hemoglobin level and appropriate intake of meat and poultry were reported among a significant majority of the studied sample (96.0%, 93.9% and 94.9%, respectively), reflecting a good adherence to antenatal care along with good protein intake among the studied sample. Meanwhile, there is a large proportion of participating women reporting adequate whole grain intake (72.5%), satisfactory dairy product intake (74.7%), careful intake of packaged food (74.6%) and only 62.3% reported adequate intake of fruits and vegetables. On the other hand, some concerning figures are found among the studied sample, such as the low prevalence of proper sun exposure (24.6%) and insufficient fish intake (23.9%), which identify some areas for potential improvements and interventions among the Saudi population. The median NRS score was 6 and the IQR was 5–7 and the majority showed low nutritional risk scores (89.1%).

Table 3 presents a comparison of FIGO nutritional elements across different stages of pregnancy in the study sample. No significant associations were found between the stage of pregnancy and nutritional habits. However, a higher frequency of meat and poultry intake was reported among participants in their third trimester (*p* = 0.01).

Table 4 illustrates the relationship between FFQ, NRS and sociodemographic and clinical variables. A total of 62 (10.9%) participants had high-risk NRS scores and 173 (30.4%) had poor-quality FFQ scores. There was no association between poor scores and any of the studied variables or outcomes; however, a significantly higher number of women with GDM had good-quality FFQ scores.

### Gestational Weight Gain Among Participants at Term

Of the 570 participants, 302 (53.0%) were at term, with a mean pre-pregnancy BMI of 30.58 ± 5.28 kg/m^2^ and the mean ± SD of GWG for the study population was 8.88 ± 5.15 kg.

**Figure 2:**
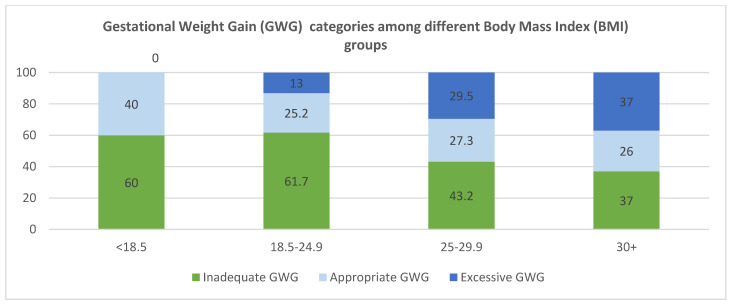
Percentage of gestational weight gain (GWG) according to IOM among various pre-pregnancy Body Mass Index (BMI) groups.

According to IOM guidelines, only 26.3% (*n* = 74) of participants achieved adequate gestational weight gain (GWG), while nearly half (49.5%, *n* = 139) showed inadequate GWG and 24.2% (*n* = 86) exceeded recommendations (Figure 2). Notably, inadequate GWG was most prevalent among underweight women (60%), followed by overweight (43.2%) and obese (37%) participants. Conversely, excessive GWG was observed in 29.5% of overweight and 37% of obese women, revealing distinct weight gain patterns across BMI categories.

**Figure 3:**
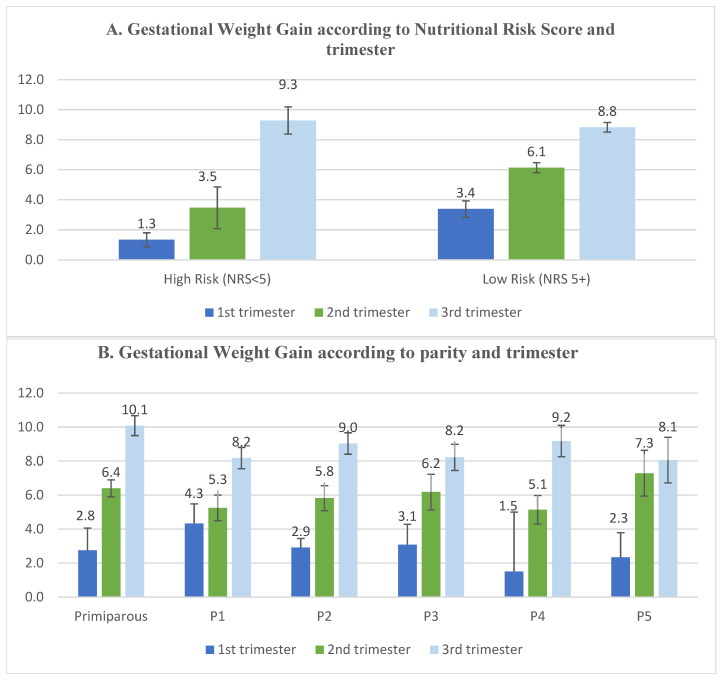
(**A**,**B**): Mean Gestational Weight Gain (in Kg) across various Nutritional Risk Scores (NRS), parity, and pregnancy trimester. Significant difference in GWG was defined among different trimesters (*p* < 0.05), no significant difference in GWG across different parities or nutritional scores.

The analysis revealed significant differences in GWG across pre-pregnancy BMI categories (*p* < 0.01). Women with normal BMI (18.5–24.9 kg/m^2^) showed the highest median GWG (8 kg, IQR: 5–12 kg), while those with obesity (BMI ≥ 30 kg/m^2^) had the lowest median gain (5 kg, IQR: 2–10 kg). However, neither parity nor nutritional scores significantly influenced GWG (Table 5), as illustrated in Figure 3A,B.

The generalized linear model (Table 6) identified pregnancy trimester as the only significant predictor of GWG among all tested variables, where the estimated marginal means of GWG in the first trimester was (3.9–6.5 Kg), second trimester (5.7–8.8 Kg), and third trimester (7.9–11.9 Kg).

## 4. Discussion

The current study used the FIGO nutrition checklist to investigate diet quality and nutritional risk factors among mothers attending antenatal care in Saudi Arabia. The results revealed that 96% of participants had at least one nutritional risk, with the median FIGO diet quality score of 6.0 (IQR: 5–7). The least reported positive response was the response to fish consumption, proper sunlight exposure, followed by insufficient intake of fruit and vegetables. Additionally, 10% of participants had poor scores on the FFQ, while 30% were classified as high-risk based on the NRS scale. Notably, poor nutritional scores were not associated with any clinical or socioeconomical variables, though women with GDM unexpectedly exhibited better dietary scores compared to others.

Consistent with our results, studies across diverse populations have similarly identified a high prevalence of nutritional risk among pregnant women when using the FIGO nutrition checklist [22,23]. This tool is proved to be comparable to other well recognized and validated nutritional assessment tools for pregnant women [24]. Its simplicity and time-efficiency make it particularly suitable for routine antenatal care, serving both as a practical screening instrument and as a foundation for targeted dietary counseling and education to address nutritional gaps [22,25].

While 30% of participants in our study had NRS below five—which is lower than reported in the Greek population [23]—this still highlights the critical need for systematic nutritional screening during pregnancy in Saudi Arabia. The most significant dietary gaps identified were insufficient consumption of fruits, vegetables, and fish, along with inadequate sunlight exposure. A diet rich in fruits, vegetables, and fish is crucial for both maternal health [26,27] and offspring development [28]. Fish provides essential omega-3 fatty acids, the deficiency of which has been associated with preterm birth, perinatal mortality, and admission to intensive neonatal care unit, as reported by the latest published Cochrane systematic review [29]. Similarly, inadequate vegetable and fruit intake during pregnancy increase the maternal risk of GDM [30] and hypertensive disorders [31], in addition to the increased risk of low birth weight and shorter newborn length [32,33].

Insufficient sunlight exposure, commonly observed in Saudi Arabia and some other countries due to cultural clothing and predominantly indoor lifestyle, is a well-established risk factor for vitamin D deficiency [34]. Studies from Saudi Arabia reported alarmingly high rates of this deficiency, affecting over 80% of pregnant women [34,35]. Vitamin D deficiency carries significant clinical implications, being associated with GDM [36] and miscarriage [37]. These findings underscore the critical need for systematic nutritional screening, targeted counseling, and appropriate micronutrient supplementation programs during the antenatal period.

While prior research has identified several risk factors associated with poor nutritional quality—including pregnancy trimester, hyperemesis gravidarum [23], and low socioeconomic status [38]—our results found no significant link between these clinical or sociodemographic factors and suboptimal nutritional scores. This discrepancy may be partially attributable to the unique characteristics of our study population, where 80% of participants fell into the high-income bracket. Additionally, conditions such as hyperemesis gravidarum were relatively uncommon in our cohort (Table 1), which may further explain the absence of these previously reported associations.

Interestingly, women with GDM demonstrated significantly better nutritional scores compared to other participants. This finding likely reflects their increased engagement with healthcare providers due to being high-risk pregnancies, resulting in more intensive nutritional monitoring and counseling.

Our study revealed no association between GWG and maternal nutrition scores. These results may stem from the FIGO assessment tool’s design, which does not evaluate key dietary components known to influence GWG—particularly energy intake, carbohydrates, and sugar consumption. These specific nutritional factors have been previously established as direct correlates of GWG in other studies [39].

Consistent with well-established trends, our study demonstrated an inverse relationship between GWG and pre-pregnancy BMI (Table 5), however, only 24% of the mothers in this study exceeded the GWG recommended by IOM (Figure 3), this proportion is almost 50% less than the proportion of 47% of mothers with excessive GWG reported in other population [40]. Inadequate GWG during pregnancy is consistently linked to certain psycho-social factors such as economic status, age group and body image dissatisfaction [41]. Unfortunately, Saudi-specific studies examining these associations remain notably absent.

Given the intricate relationship between pre-pregnancy weight and GWG [42,43], our findings suggest that GWG is unlikely to be the main precursor of prepregnancy obesity among Saudi women which was noticed in nearly 60% of the women in this study (Table 1).

### 4.1. Implication to Practice and Research

#### The Results of This Study Suggest

1. There is an urgent need for the implementation of nutritional assessment and counselling program for pregnant Saudi mothers, preferably to cover preconception, antenatal and postnatal periods, considering that 96% of the study cohort had at least one nutritional deficiency risk.
2. Given the unique nutritional deficiency profile of Saudi mothers, healthcare providers should have the knowledge to manage all variations of maternal nutritional deficiencies and to provide counselling and advice.
3. Estimation of vitamin D level should be included in the antenatal screening tests in Saudi Arabia and guidelines for replacement therapy should be available, considering that exposure to sunlight is one of the least positive responses in this study.
4. Regular monitoring of GWG during pregnancy and counselling mothers to achieve proper GWG based on their BMI should be enforced considering that 50% of the mothers in this study group had inadequate GWG.
5. Further research should be directed towards the investigation of the effects of nutritional deficiencies on the maternal and fetal outcomes of pregnancy in Saudi Arabia, especially vitamin D deficiency.
6. Investigation of the associations and the outcomes of inadequate GWG among Saudi women.
7. It is important to explore the opinions of the mothers and their families about healthy nutrition during pregnancy, which will form a solid base for successful evidence-based interventions and will place the mother at the center of care.

## 5. Strengths and Limitations

This study is the first in Saudi Arabia to map the nutritional profile of pregnant women using the FIGO assessment tool, and it has highlighted important areas for maternal healthcare provision improvement. We acknowledge certain limitations in this study, including the relatively small number of participants in each trimester of pregnancy, which may have introduced imprecision in the results. Furthermore, the study was conducted in one center and in one city, which limits generalization of the results to other parts of the country. However, our results will be an important base for other researchers to explore this field further.

Further investigation into pregnancy outcomes among mothers whose nutritional status was assessed using the FIGO tool during gestation would provide valuable insights into its predictive capacity for various obstetric outcomes. Such research would significantly enhance our understanding of the tool’s clinical utility in antenatal care.

The FIGO nutritional tool used in this study does not assess maternal energy intake or consumption, which are important factors in the assessment of the relationship between GWG and nutrition and could have explained the inadequate GWG observed in almost 50% of the participants. In addition, the tool does not cater to women with certain conditions, such as those with lactose intolerance, irritable bowel syndrome, or those who are vegetarian.

## 6. Conclusions

Although poor nutritional quality and high nutritional risk are relatively uncommon among Saudi women, the prevalence rates remain consistent across all sociodemographic groups. This suggests widespread, uniform patterns of suboptimal dietary habits within the community. While GWG was not affected by nutritional status or parity of the participants, nearly half of the participants had inadequate GWG, particularly those with a low pre-pregnancy BMI.

## Figures and Tables

**Table 1:** Characteristics of the studied sample.

		Frequency (N)	Percentage (%)
**Age (years)**	less than 20	5	0.9
	21–35	411	72.4
	36+	152	26.8
Education	School	182	31.9
	University	341	59.8
	Postgraduate	47	8.2
Job	Student	22	3.9
	Housewife	374	65.8
	Employee	172	30.3
Family Income	Enough and save	125	21.9
	Enough	336	58.9
	Not enough	104	18.2
	In debt	5	0.9
Trimester	1st trimester	78	13.7
	2nd trimester	190	33.3
	3rd trimester	302	53.0
Pre-pregnancy BMI (kg/m^2^)	<18.5	20	3.7
	18.5–24.9	210	38.4
	25–29.9	176	32.2
	30+	141	25.8
Diabetes Mellitus	no	548	96.1
	yes	22	3.9
Gestational Diabetes	no	502	88.1
	yes	68	11.9
Hypertension	no	558	97.9
	yes	12	2.1
Hyperemesis gravida	no	563	98.8
	yes	7	1.2
Data are presented as frequency and percentages			

**Table 2:** Distribution of FIGO elements in the studied sample.

	Positive Answers	
	Frequency (N)	Percentage (%)
**Diet Quality Score (FFQ)**		
Meat and poultry 2–3 per week	541	94.9
Fruits and vegetable 2–3 daily	355	62.3
Fish 1–2 weekly	136	23.9
Dairy products daily	426	74.7
Whole grain carbs ≥ 1/day	413	72.5
Packaged food <5 times per week	425	74.6
No negative answers	1	0.2
One or more negative answers	569	99.8
Poor quality FFQ (≤3)	173	30.4
Good quality FFQ (>3)	397	69.6
**Food Supplements**		
Proper intake of folic acid	547	96.0
Enough sun exposure	140	24.6
No anemia	535	93.9
No negative answers	0	0.0
One or more negative answers	570	100.0
**FIGO—overall nutritional risk score (NRS)** (Median, Interquartile Range)	(6, 5–7)	
High Risk (NRS < 5)	62	10.9
Low Risk (NRS ≥ 5)	508	89.1

FFQ: food frequency quality, NRS: Nutritional Risk Score.

**Table 3:** FIGO elements among participants according to the stage of pregnancy.

	Trimester						*p*-Value
	1st Trimester		2nd Trimester		3rd Trimester		
	N	%	N	%	N	%	
**Food Frequency items (FFQ)**							
Meat and poultry 2–3 per week	69	88.5	180	94.7	292	96.7	0.01
Fruits and vegetables 2–3 daily	43	55.1	122	64.2	190	62.9	0.36
Fish 1–2 weekly	16	20.5	43	22.6	77	25.5	0.58
dairy products daily	59	75.6	141	74.2	226	74.8	0.97
Whole grain carbs ≥ 1/day	55	70.5	140	73.7	218	72.2	0.86
packaged food <5 times per week	60	76.9	137	72.1	228	75.5	0.62
FFQ (>3 good quality)	47	60.3	135	71.1	215	71.2	0.15
FFQ (≤3 poor quality)	31	39.7	55	28.9	87	28.8	
**Food Supplements**							
Folic acid intake	71	91.0	185	97.4	291	96.4	0.05
Good sun exposure	18	23.1	57	30.0	65	21.5	0.09
Normal hemoglobin	75	96.2	177	93.2	283	93.7	0.64
**Total FIGO Score**							
High Risk NRS (<5)	9	11.5	17	8.9	36	11.9	0.58
Low Risk NRS (≥5)	69	88.5	173	91.1	266	88.1	
GWG (in Kg) (mean ± SD)	3.14 ± 4.27		5.92 ± 4.43		8.88 ± 5.10		<0.01

FFQ: food frequency quality, NRS: Nutritional Risk Score, GWG: Gestational Weight Gain.

**Table 4:** Food Frequency Quality (FFQ) and Nutritional Risk Score (NRS) among different subgroups.

		FFQ		NRS	
		Poor Quality (≤3) N (%)	Good Quality (>3) N %	High Risk (<5) N (%)	Low Risk (≥5) N (%)
Age (years)	less than 20	2 (40.0)	3 (60.0)	0 (0.0)	5 (100)
	21–35	132 (32.1)	279 (67.9)	50 (12.2)	361 (87.8)
	36+	38 (25.0)	114 (75.0)	12 (7.9)	140 (92.1)
	*p*-value	0.23		0.26	
Education attainment	School	52 (28.6)	130 (71.4)	20 (11.0)	162 (89.0)
	University	108 (31.7)	233 (68.3)	38 (11.1)	303 (88.9)
	Postgraduate	13 (27.7)	34 (72.3)	4 (8.5)	43 (91.5)
	*p*-value	0.96		0.86	
Occupation	Student	8 (36.4)	14 (63.6)	3 (13.6)	19 (86.4)
	Housewife	111 (29.7)	263 (70.3)	41 (11.0)	333 (89.0)
	Employee	52 (30.2)	120 (69.8)	16 (9.3)	156 (90.7)
	*p*-value	0.81		0.75	
Family income	Enough and save	39 (31.2)	86 (68.8)	17 (13.7)	108 (86.4)
	Enough	100 (29.8)	236 (70.2)	28 (8.3)	308 (91.7)
	Not enough	33 (31.7)	71 (68.3)	17 (16.3)	87 (83.7)
	In debt	1 (20.0)	4 (80.0)	0 (0.0)	5 (100.0)
	*p*-value	0.93		0.07	
Pre-pregnancy BMI (kg/m^2^)	<18.5	6 (30.0)	14 (70.0)	2 (10.0)	18 (90.0)
	18.5–24.9	64 (30.5)	146 (69.5)	22 (10.5)	188 (89.5)
	25–29.9	52 (29.5)	124 (70.5)	15 (8.5)	161 (91.5)
	30+	41 (29.1)	100 (70.9)	17 (12.1)	124 (87.9)
	*p*-value	0.99		0.78	
GWG	Adequate	36 (27.9)	93 (72.1)	10 (7.8)	119 (92.2)
	Below	102 (31.4)	223 (68.6)	39 (12.0)	286 (88.0)
	Above	25 (29.1)	61 (70.9)	7 (8.1)	79 (91.9)
	*p*-value	0.77		0.31	
Diabetes Mellitus	No	166 (30.3)	382 (69.7)	60 (10.9)	488 (89.1)
	Yes	7 (31.8)	15 (68.2)	3 (4.4)	65 (95.6)
	*p*-value	0.89		0.78	
Gestational Diabetes	No	163 (32.5)	339 (67.5)	59 (11.8)	443 (88.2)
	Yes	10 (14.7)	58 (85.3)	3 (4.4)	65 (95.6)
	*p*-value	<0.01		0.06	
Hypertension	No	168 (31.0)	390 (69.9)	59 (10.6)	499 (89.4)
	Yes	5 (41.7)	7 (58.3)	3 (25.0)	9 (75.0)
	*p*-value	0.39		0.11	

BMI: Body Mass Index, FFQ: Food Frequency quality, NRS: Nutritional Risk Score, GWG: Gestational Weight Gain.

**Table 5:** Gestational weight gain at the term among different groups of study participants.

		Gestational Weight Gain in Kg Median (IQR)	*p*-Value
**Trimester**	First trimester	2 (0–5)	<0.01
	Second trimester	6 (3–9)	
	Third trimester	9 (5–12)	
**Pre-pregnancy BMI (kg/m^2^)**	<18.5	5 (3–11)	<0.01
	18.5–24.9	8 (5–12)	
	25–29.9	6 (3–10)	
	30+	5 (2–10)	
**FIGO-NRS**	High risk	5.5 (2–11)	0.34
	Low risk	6 (4–10)	

IQR: Interquartile range, BMI: Body Mass Index, NRS: Nutritional Risk Score. Kruskall–Wallis test and Mann–Whitney test were used.

**Table 6:** Generalized linear model of Gestational Weight Gain (GWG) and FIGO scores adjusted for age, trimester of pregnancy, parity, Gestational Diabetes and Hypertensive disorders during pregnancy.

Model	Unstandardized Coefficients		Wald Chi-Square	*p*-Value
	B	Std. Error		
**(Intercept)**	1.79	0.21	73.5	**<0.01**
**Parity**	0.15	0.01	1.17	0.28
**Age**	−0.01	0.005	4.01	0.05
**Trimester**				
**First ^#^**	-	-	-	**<0.01**
**Second**	0.35	0.09	14.76	
**Third**	0.67	0.09	59.4	
**Total FIGO (NRS)**	0.02	0.02	0.69	0.49
**GDM**	0.03	0.08	0.11	0.74
**HTN**	0.08	0.19	0.17	0.68
**Estimated Marginal means of Gestational Weight Gain in different trimesters**				
	**Mean GWG**	**Standard Error**	**95% Wald Confidence Interval**	
**Third Trimester**	9.77	1.01	7.98–11.98	
**Second Trimester**	7.13	0.75	5.80–8.76	
**First Trimester**	5.03	0.64	3.91–6.47	

^#^ reference group, NRS: Nutritional Risk Score, GDM: Gestational Diabetes, HTN: hypertensive disorders during pregnancy. Likelihood Ratio Chi-square = 71.52, df = 7, *p*-value < 0.001. Covariates appearing in the model are fixed at the following values: parity = 1.92, age = 31.9, FIGO total score = 6.12.

## Data Availability

Data from this study is available to researchers upon request and approval of Institutional Review Board at Princess Nourah bint Abdulrahaman University (irb@pnu.edu.sa). The request and approval of data sharing are independent from the research team.
